# The Possibility of Ceftolozane/Tazobactam-Resistant Pseudomonas aeruginosa Emergence After Two Days of Antibiotic Therapy: A Case Report

**DOI:** 10.7759/cureus.79207

**Published:** 2025-02-18

**Authors:** Yoshihide Hioki, Takehiro Hashimoto, Kazufumi Hiramatsu, Kosaku Komiya

**Affiliations:** 1 Department of Respiratory Medicine and Infectious Diseases, Oita University Faculty of Medicine, Yufu, JPN

**Keywords:** antibiotic exposure, antimicrobial resistance, p. aeruginosa, pneumonia, tazobactam/ceftolozane

## Abstract

Although antibiotic use is known to induce antimicrobial resistance, the duration of exposure necessary for resistance development remains uncertain. In this case, a patient was initially treated with tazobactam/piperacillin (TAZ/PIPC) for bacterial pneumonia. When the treatment proved ineffective, the regimen was switched to tazobactam/ceftolozane (TAZ/CTLZ) after confirming that the *Pseudomonas aeruginosa* isolated at admission was susceptible to TAZ/CTLZ. Although the patient’s symptoms initially improved, pneumonia exacerbation occurred 10 days after the initiation of TAZ/CTLZ. Drug susceptibility testing in *P. aeruginosa* isolated on the second day of TAZ/CTLZ treatment revealed resistance to the antibiotics. Genetic analysis using the polymerase chain reaction (PCR)-based open reading frame typing method demonstrated that the *P. aeruginosa* strains isolated before and after TAZ/CTLZ treatment were genetically identical. This case highlights the possibility of TAZ/CTLZ-resistant *P. aeruginosa* emerging after only two days of antibiotic exposure.

## Introduction

Tazobactam/ceftolozane (TAZ/CTLZ) exhibits potent antibacterial activity against aerobic Gram-negative bacilli, including extended-spectrum beta-lactamase (ESBL)-producing bacteria and *Pseudomonas aeruginosa*. It is recommended as an empiric regimen for patients with hospital-acquired pneumonia (HAP) within the spectrum of respiratory infectious diseases [1,2]. TAZ/CTLZ has demonstrated non-inferiority to meropenem (MEPM) in the treatment of patients with HAP [3]. Moreover, TAZ/CTLZ is less susceptible to major drug resistance mechanisms, such as outer membrane modifications and drug efflux pumps [4,5]. Tazobactam effectively inhibits ESBL, while ceftolozane remains stable against AmpC beta-lactamases [6,7]. Furthermore, TAZ/CTLZ is less likely to induce drug resistance in patients with HAP caused by *P. aeruginosa* compared to MEPM treatment [4]. Here, we report a case in which *P. aeruginosa* resistant to TAZ/CTLZ was isolated from sputum after only two days of treatment, despite being susceptible prior to the intervention. Genetic analysis using the polymerase chain reaction (PCR)-based open reading frame typing (POT) method suggested that the strains isolated before and after treatment were identical. This finding raises the possibility that short-term exposure to TAZ/CTLZ may contribute to the development of resistance, warranting further investigation. This case was previously presented as a meeting abstract at the 94th Academic Meeting of the Western Japan Branch, The Japanese Association for Infectious Diseases on November 14, 2024.

## Case presentation

A 72-year-old woman with rheumatoid arthritis-associated interstitial pneumonia presented to the hospital with complaints of dyspnea. She had been taking 40 mg of prednisolone daily for the management of rheumatoid arthritis. Rheumatoid arthritis was diagnosed eight years ago, and although methotrexate and other immunosuppressive agents were attempted, they were discontinued due to adverse effects. She had been taking oral trimethoprim/sulfamethoxazole (400 mg sulfamethoxazole /80 mg trimethoprim) once daily for Pneumocystis pneumonia (PCP) prophylaxis. There was no history of antibiotic use in the past year.

**Figure 1:**
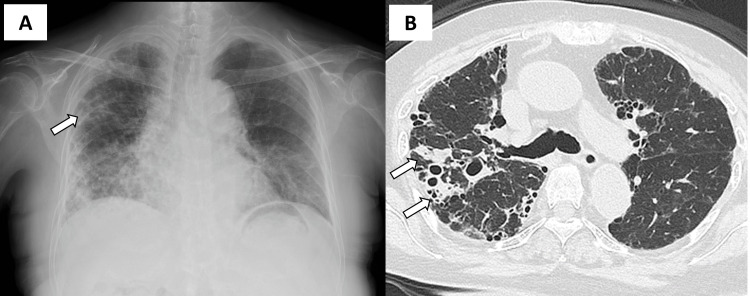
Chest X-ray and computed tomography images at the time of hospitalization The chest X-ray revealed newly developed consolidation in the right upper lung field (arrow) accompanied by pre-existing reticular shadows in the lower bilateral lung fields (A). Chest computed tomography (CT) demonstrated consolidation with bronchial wall thickening in the right upper lobe (arrow) (B).

High-resolution computed tomography (HRCT) of the chest revealed newly developed ground-glass opacities in the upper lobe of the right lung, leading to her referral to our hospital for further evaluation. On physical examination, her vital signs included a body temperature of 36.5°C, a SpO₂ of 88% on room air, a respiratory rate of 21 breaths per minute, blood pressure of 110/79 mmHg, and a heart rate of 103 beats per minute. Notable findings included a moon-shaped face, bilateral lower leg edema, and fine crackles auscultated in the bilateral lower lung fields. Laboratory investigations revealed elevated levels of C-reactive protein (8.35 mg/dL), white blood cell count (9,690/μL), and lactate dehydrogenase (332 IU/L), while hemoglobin (13.9 g/dL), blood urea nitrogen (18.9 mg/dL), and serum creatinine (0.45 mg/dL) were within normal limits. Tests for antibodies specific to collagen vascular diseases were negative. A chest X-ray obtained at our hospital revealed newly developed pulmonary infiltration in the right upper lung field accompanied by pre-existing reticular shadows in the lower bilateral lung fields (Figure 1A). Chest computed tomography demonstrated consolidation with bronchial wall thickening in the right upper lobe (Figure 1B).

Given the possibility of a bacterial infection complicating rheumatoid arthritis-associated interstitial pneumonia, antimicrobial therapy with tazobactam/piperacillin (TAZ/PIPC) was initiated at a dose of 4.5 g, administered four times daily. This decision was based on a purulent sputum smear, which revealed Gram-negative bacilli morphologically resembling *P. aeruginosa*. As TAZ/PIPC proved ineffective, the antibiotic regimen was switched to tazobactam/ceftolozane (TAZ/CTLZ) at 3 g, administered three times daily on the fifth day of admission. This decision was based on its potent antimicrobial activity against *P. aeruginosa* isolated from sputum at admission, with results indicating that the minimum inhibitory concentration (MIC) of TAZ/CTLZ was lower than that of other agents, including TAZ/PIPC, ceftazidime, and amikacin. Following the switch to TAZ/CTLZ, the patient’s respiratory symptoms and inflammatory markers gradually improved. However, on the 14th day of admission, her wet cough worsened, and inflammatory markers increased. Drug susceptibility testing of *P. aeruginosa* isolated from purulent sputum on the second day of TAZ/CTLZ administration revealed resistance to TAZ/CTLZ, although susceptibility to meropenem (MEPM) and amikacin was preserved (Table 1) [8]. Given that MEPM had the lowest MIC, subsequent treatment with MEPM (1 g, three times daily) led to significant symptomatic improvement, with normalization of inflammatory markers. The patient was successfully discharged on the 40th day of hospitalization. The *P. aeruginosa* isolates obtained at admission and on the second day of TAZ/CTLZ administration were both of the mucoid phenotype. The antimicrobial susceptibility of mucoid *P. aeruginosa* can sometimes be overestimated due to insufficient bacterial growth at 24 hours [9]. To ensure accurate susceptibility testing, our hospital follows a 48-hour incubation protocol for bacterial cultures of the mucoid phenotype in accordance with the Manual of Clinical Microbiology, 13th ed. [10]. However, this incubation protocol does not conform to the Clinical and Laboratory Standards Institute (CLSI) M07 guidelines.

**Table 1:** Susceptibility results against Pseudomonas aeruginosa isolated on the day of admission and after two days of treatment with TAZ/CTLZ The minimum inhibitory concentration (MIC) breakpoint is referenced from Performance standards for antimicrobial susceptibility testing. In Clinical and Laboratory Standards Institute (CLSI) supplement M100, 34th. Clinical and Laboratory Standards Institute, Wayne, PA [8]. Since the isolate was of the mucoid phenotype, the incubation period was extended to 48 hours [10]. AMK: amikacin, AZT: aztreonam, CAZ: ceftazidime, CFPM: cefepime, CPFX: ciprofloxacin, MEPM: meropenem, MIC: minimum inhibitory concentration, PIPC: piperacillin, TAZ/CTLZ: tazobactam/ceftrozane, TAZ/PIPC: tazobactam/piperacillin

Timing	The day of admission	After two days of treatment with TAZ/CTLZ	MIC breakpoint (mg/L)		
			Sensitive	Intermediate	Resistance
PIPC	8	>128	≤16	32	≥64
TAZ/PIPC	8	>128	≤16 / 4	32 / 4	≥64 /4
TAZ/CTLZ	2	>64	≤4 / 4	8 / 4	≥16 / 4
CAZ	8	>64	≤8	16	≥32
CFPM	16	>64	≤8	16	≥32
AZT	16	>64	≤8	16	≥32
MEPM	≤0.5	≤0.5	≤2	4	≥8
AMK	4	8	≤16	32	≥64
CPFX	1	4	≤0.5	1	≥2

**Figure 2:**
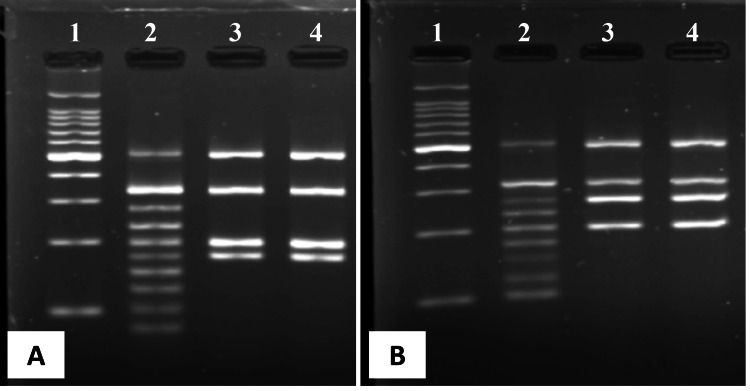
Results of PCR-based open reading frame typing. The test for Pseudomonas aeruginosa included two reaction mixtures (A and B). Lanes 1 and 2 represent the DNA ladder and positive control, respectively. Lane 3 corresponds to the strain isolated at admission, while Lane 4 corresponds to the strain isolated two days after the initiation of tazobactam/ceftolozane treatment. PCR: Polymerase chain reaction.

To assess their genetic identity, we compared the *P. aeruginosa* strains isolated at admission and two days after the initiation of TAZ/CTLZ treatment using POT with the Cica Geneus Pseudo POT kit (Kanto Chemicals, Tokyo, Japan). POT is a genotyping method that identifies bacterial isolates by detecting variations in open reading frames (ORFs) using multiplex PCR [11]. This system for *P. aeruginosa* is designed to analyze 17 ORFs, enabling strain identification based on their unique genetic patterns. In this case, the band patterns of the strains were identical, suggesting that the *P. aeruginosa* strains isolated before and after TAZ/CTLZ treatment were genetically identical (Figure 2), despite the lack of confirmation through multilocus sequence typing (MLST) or whole-genome sequencing (WGS).

## Discussion

In the present case, TAZ/CTLZ-resistant *P. aeruginosa* was isolated after two days of treatment with TAZ/CTLZ, while the strain isolated prior to treatment was susceptible. Genetic analysis using the POT method confirmed that the two strains were genetically identical. The POT method is widely utilized for strain identification, offering comparable discriminatory power to pulsed-field gel electrophoresis (PFGE) and MLST, while being faster and simpler to perform [12]. The Simpson’s index, which measures the probability that two unrelated strains will be classified as distinct using the POT method, was 0.99 for the two isolates of *P. aeruginosa*, indicating a 99% discriminatory ability [13].

As part of a subgroup analysis from a randomized controlled trial demonstrating the non-inferiority of TAZ/CTLZ compared to MEPM for HAP [3], the administration of TAZ/CTLZ was reported not to induce resistance to TAZ/CTLZ in *P. aeruginosa* [4]. In this analysis, among 59 isolates of *P. aeruginosa*, three strains were found to be resistant to TAZ/CTLZ following its administration. However, MLST and WGS identified differences in resistance gene profiles, suggesting the presence of novel *P. aeruginosa* infections. A multicenter retrospective cohort study conducted in the United States evaluated TAZ/CTLZ resistance in organisms detected before and after treatment in patients with multidrug-resistant Gram-negative rod infections [14]. Approximately 10% of strains were reported to develop resistance to TAZ/CTLZ, with the shortest duration for resistance emergence estimated at three days. However, this study did not assess whether the strains were genetically identical before and after treatment. In the current case, based on the results of POT, the emergence of resistance following short-term exposure to TAZ/CTLZ cannot be ruled out.

Meanwhile, it is important to consider the possibility that *P. aeruginosa* did not develop resistance as a direct result of the current administration of TAZ/CTLZ. Even within the same organism isolated from a single host, individual colonies may exhibit varying drug susceptibilities. The patient had a history of repeated respiratory tract infections and rheumatoid arthritis-associated chronic bronchiolitis, both of which could have contributed to the emergence of TAZ/CTLZ-resistant *P. aeruginosa* due to prior antibiotic exposure. The concept of "heteroresistance" has gained increasing recognition in recent years [15,16]. Heteroresistance refers to the presence of subpopulations within a primary bacterial strain that exhibits significantly reduced antimicrobial susceptibility compared to the main population [17]. Indeed, studies have reported varying drug susceptibilities of *P. aeruginosa* within the same patient, particularly in cases of cystic fibrosis. For example, Monogue et al. demonstrated that *P. aeruginosa* can form morphologically and genetically distinct colonies with differing antimicrobial susceptibility patterns [18]. In the current case, TAZ/CTLZ treatment appeared effective during the initial phase of treatment. It is plausible that subpopulations of *P. aeruginosa* with varying drug susceptibilities coexisted, and the TAZ/CTLZ-susceptible subpopulation was eradicated early in the course of treatment. Antibiotic selection was guided by the MIC of *P. aeruginosa* isolated from the patient's sputum. However, it is crucial to acknowledge that the assumption that an antimicrobial agent with a lower MIC necessarily exhibits superior bactericidal activity in vivo is not scientifically valid despite this principle often influencing clinical decision-making.

This case highlights two key possibilities: the emergence of drug resistance following short-term exposure to TAZ/CTLZ and the presence of heteroresistance in *P. aeruginosa*. A notable strength of this report is the confirmation of genetic similarity between isolates obtained before and after antibiotic treatment using the POT method. However, several limitations warrant discussion. First, the possibility of a concomitant bacterial infection alongside *P. aeruginosa* must be considered. As described, the patient’s symptoms initially improved with TAZ/CTLZ treatment, despite the *P. aeruginosa* isolated prior to treatment later being found resistant to TAZ/CTLZ. While this phenomenon may be explained by heteroresistance, it is also possible that the treatment targeted a coexisting bacterial infection susceptible to TAZ/CTLZ. Second, the limitations of bacterial identification using the POT method should be acknowledged. Although POT provides similar discriminatory power to PFGE and MLST, with a high Simpson’s index of 0.99, there remains a 1% risk of misidentifying distinct strains as identical [13]. Therefore, we cannot entirely rule out the possibility of minute genetic variations among the strains considered 'genetically identical' in this study. The POT method classifies strains based on ORF patterns but does not directly assess mutations or changes in the expression of drug-resistance genes. Since we did not conduct detailed genetic mutation analysis using WGS or MLST, it remains unclear whether the observed resistance resulted from newly acquired mutations or the selective proliferation of pre-existing resistant subpopulations. Furthermore, as previously discussed, TAZ/CTLZ-resistant subpopulations of *P. aeruginosa* may have been present prior to treatment, with TAZ/CTLZ administration selectively eliminating the susceptible population, thereby allowing the resistant subpopulation to predominate. However, in this study, susceptibility testing was performed on a single colony from each time point, limiting our ability to fully assess heteroresistance.

Future studies should incorporate multi-colony analyses and population profiling to better characterize this phenomenon. Finally, the isolated *P. aeruginosa* in this case exhibited a mucoid phenotype. The antimicrobial susceptibility of mucoid *P. aeruginosa* may be inaccurately estimated due to insufficient growth at 24 hours [9]. While our hospital adheres to a 48-hour incubation protocol for bacterial cultures in accordance with the Manual of Clinical Microbiology, 13th ed. [10], the precise accuracy of this approach remains uncertain.

## Conclusions

This case report highlights two key possibilities: the emergence of TAZ/CTLZ resistance after just two days of exposure and the presence of heteroresistance, where *P. aeruginosa* subpopulations exhibit varying susceptibility to TAZ/CTLZ prior to antibiotic treatment. In either scenario, the findings underscore the importance of recognizing that a single-point drug susceptibility test result may be insufficient. Repeated susceptibility testing should be considered, even for the same pathogen isolate, when clinical improvement is not observed as expected during antibiotic therapy.
